# Semi‐Flooded Sulfur Cathode with Ultralean Absorbed Electrolyte in Li–S Battery

**DOI:** 10.1002/advs.201903168

**Published:** 2020-03-18

**Authors:** Yong Xie, Guoyu Pan, Qiang Jin, Xiaoqun Qi, Tan Wang, Wei Li, Hui Xu, Yuheng Zheng, Sa Li, Long Qie, Yunhui Huang, Ju Li

**Affiliations:** ^1^ Institute of New Energy for Vehicles School of Materials Science and Engineering Tongji University Shanghai 201804 China; ^2^ Department of Nuclear Science and Engineering and Department of Materials Science and Engineering Massachusetts Institute of Technology Cambridge MA 02139 USA

**Keywords:** canal–capillary microstructures, fill factor, high‐loading electrodes, lean electrolytes, lithium–sulfur batteries

## Abstract

Lean electrolyte (small E/S ratio) is urgently needed to achieve high practical energy densities in Li–S batteries, but there is a distinction between the cathode's absorbed electrolyte (AE) which is cathode‐intrinsic and total added electrolyte (E) which depends on cell geometry. While total pore volume in sulfur cathodes affects AE/S and performance, it is shown here that pore morphology, size, connectivity, and fill factor all matter. Compared to conventional thermally dried sulfur cathodes that usually render “open lakes” and closed pores, a freeze‐dried and compressed (FDS‐C) sulfur cathode is developed with a canal‐capillary pore structure, which exhibits high mean performance and greatly reduces cell‐to‐cell variation, even at high sulfur loading (14.2 mg cm^−2^) and ultralean electrolyte condition (AE/S = 1.2 µL mg^−1^). Interestingly, as AE/S is swept from 2 to 1.2 µL mg^−1^, the electrode pores go from fully flooded to semi‐flooded, and the coin cell still maintains function until (AE/S)_min_ ≈ 1.2 µL mg^−1^ is reached. When scaled up to Ah‐level pouch cells, the full‐cell energy density can reach 481 Wh kg^−1^ as its E/S ≈ AE/S ratio can be reduced to 1.2 µL mg^−1^, proving high‐performance pouch cells can actually be working in the ultralean, semi‐flooded regime.

## Graphical abstract

Unprecedented cathode performance and performance consistency are achieved using raw sulfur without nanostructured host (hostless), Ketjen Black as the only conductive agent (no carbon nanotube, graphene, etc.) and liquid electrolyte as lean as 1.2 µL mg^−1^, by pore microstructure engineering with freeze‐drying. Pouch cell and coin cell results are transferrable if absorbed electrolyte is used instead of total electrolyte.

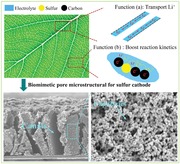

## 1 Introduction

Lithium–sulfur (Li–S) battery chemistry has a high theoretical energy density of 2600 Wh kg^−1^ based on the multielectron anion‐redox S_8_ + 16 Li^+^ + 16 e^−^ ↔ 8 Li_2_S reaction, several times higher than state‐of‐art lithium‐ion batteries (LIB).^1^ However, highly porous S_8_ cathode^2^ and superabundant electrolyte (e.g., E/S ratio >10 µL mg^−1^ for coin cells and >3 µL mg^−1^ for pouch cells)^3^ are often cited in the literature to reach satisfactory sulfur utilization and cycling numbers. In contrast, E/S in LIB is only ≈0.3 µL mg^−1^.3e A high E/S drastically reduces the Li–S full‐cell gravimetric energy density.^1^, ^4^ For example, when E/S > 10 µL mg^−1^, the energy density of full‐cell cannot be more than 200 Wh kg^−1^ even with S_8_ loading of 6 mg cm^−2^, 75 wt% S_8_ in the cathode and 80% sulfur utilization (1337 mAh g^−1^), as shown in Figure S1a in the Supporting Information.^5^ Fundamentally, ether‐based liquid electrolyte phase serves two purposes in such S_8_ cathode, as illustrated in **Figure** **1**: a) it serves as the “waterways” for the long‐range transport of Li^+^ and b) it dissolves lithium polysulfide (LiPS) and boosts the redox kinetics in contact with conductive carbon black,^3^, ^6^ as local sulfur mobility (LSM)^7^ is often required to mediate the redox reaction. However, global sulfur mobility (GSM) is undesirable because it leads to sulfur crossover to the anode or layering of electronically insulating phases within the cathode.^7^, ^8^ To fulfill the above‐mentioned two electrolyte functions in S_8_ cathode, one must carefully and rationally engineer the electrolyte/electrode pore space distributions within the cathode. Inspired by the plant leaf illustrated in Figure 1, to support function (a), end‐to‐end canal “waterways” are essential for the long‐range mass transport of Li^+^ over a length scale of 10^1^ µm; On the other hand, to support function (b), multiconnected capillary network at a length scale of 10^1^–10^2^ nm are also needed because the conductive carbon nanoparticles are dispersed at such length scale, and LSM is needed at such 10^1^–10^2^ nm length scale for the solubilized LiPS to waft to the nearest conductive carbon particle to sustain redox reactions: such local consumptions also help shut down GSM and eliminate insulator‐dense‐layering (without porosity or carbon black) tendencies within the cathode.^7^, ^8^

**Figure Figure 1:**
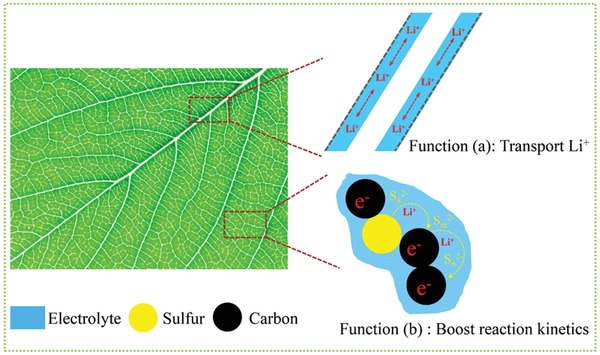
The schematics of the two basic functions of liquid electrolyte in a sulfur cathode. Function (a): serve as the “waterways” for the long‐range transport of Li^+^; Function (b): dissolve lithium polysulfides (LiPS) and boost the local redox kinetics. We believe that a biomimetic electrode pore structure (low tortuous “canals” plus multiconnected “capillaries”) is the best way to promote liquid electrolyte to serve the two functions.

Here we want to emphasize that the electrode pores are completely different from sulfur‐host pores, which were generally elaborately constructed to induce sulfur impregnation. The sulfur‐host porosity needs to be big in order to load in more sulfur.^9^ They also need to be highly tortuous and less connected in order to suppress GSM.^10^ However, these rules are not applicable to electrode pores. We found that some electrode pores need to be interconnected with low tortuosity to assist electrolyte imbibition and long‐range Li^+^ transport, and the total electrode porosity P needs to be relatively low, in order to absorb less electrolyte. There is no straightforward relation between sulfur host pores and electrode pores. The original host pores may be significantly altered by the sulfur impregnation and electrode slurrying process which disrupts the host pore connectivity and size. Indeed, in this paper we focus on so‐called host‐free sulfur electrode where raw S_8_ are directly mixed with raw Ketjen Black (KB) without elaborate nanostructured hosts. In this case, studying the electrode pore structure (not only pore size but also pore morphology, pore connectivity, and fill factor (FF)) directly is the most meaningful.

As electrolyte generally exists in the pores of the cathode, the relationship between porosity and electrolyte usage is worthy of careful discussions. In the energy density calculations,5a it has been standard practice to assume that any volume not occupied by S_8_, binder, and carbon black (i.e., total cathode porosity *P*) is fully flooded by the electrolyte. However, as we will show in this paper, conventionally prepared thermally dried sulfur (TDS) cathode usually has a large amount of close‐off pores that can serve neither function (a) nor (b) stated at the beginning (see also Figure 1), even under externally superabundant electrolyte condition. Thus, we need to distinguish between open porosity (OP) and closed porosity (CP), where P = OP + CP are dimensionless quantities that range between 0 and 100 vol%. Also, even the open pores, under very lean electrolyte condition, do not have to be completely flooded. We can define the FF to be the fraction of pore volume that is actually occupied by liquid electrolyte, so we get a relationship absorbed electrolyte (AE) = *V*_cathode_(OP × FF_OP_ + CP × FF_CP_), where AE stands for cathode's absorbed electrolyte volume [unit m^3^], FF_OP_ is the open‐pore fill factor, and FF_CP_ is the closed‐pore fill factor (as with cycling, some previously open pores can become closed, with electrolyte retained inside). The fill factor takes cue from rivers and canals in dry seasons with partially exposed river beds that would still maintain to be navigable, until a critical percolation threshold is reached.

To complicate the discussion further, especially in laboratory coin cells, the total added electrolyte (E) is not all absorbed inside the cathode, considering there is reserved electrolyte (RE) in the head spaces and wetting with the cell packaging, so we have the relationship E = RE + AE. We note that AE is more “cathode‐intrinsic,” while E also depends on factors not intrinsic to the cathode microstructure, e.g. cell size, headspace design, cell packaging material, etc. RE is not immediately useful for cathode functions (a) or (b), but acts as a reserve in case of porosity evolution and/or electrolyte consumption by the anode. The electrolyte “absorptivity” is defined as AE/E, a dimensionless number. Surprisingly, we find TDS tends to have a low AE/E ratio, due to binder evaporation‐and‐sealing from the high‐temperature solvent evaporation and cooling‐off process.^11^ So even under a superabundant electrolyte condition like E/S = 10 µL mg^−1^, the inside of TDS can still nearly be *depleted of liquid electrolyte* (i.e., AE/S is low while RE/S is high). This severely disrupts cathode function (a) and (b), leading to poor average performance and very scattered cell‐to‐cell behavioral variation, i.e., poor performance consistency, characterized by a large standard deviation in the measured cell discharge capacity from cell to cell.

In this work, to pursue practical high energy density Li–S batteries by bringing down E/S ratio while maintaining the performance consistency (e.g., reducing cell‐to‐cell variations, and not just reporting the *best* cell performance after making a batch of, say, five nominally identical cells), a simple and scalable method of lyophilization plus compression was developed to produce a stress‐relieved canal/capillary electrode microstructure. With our newly prepared freeze‐dried‐and‐compressed sulfur (FDS‐C) cathode, one can eliminate most of the “bad” porosities, leaving only the “good” canals and capillaries. FDS‐C has higher AE/E ratio than TDS, so even when E is very low under lean E/S condition, there are sufficient AE (and also with good AE morphologies) to sustain functions (a) and (b). This leads to higher sulfur utilization (814 mAh g^−1^ → 1264 mAh g^−1^) and better performance across the board than TDS, and 20 × better cell‐to‐cell performance consistency. The standard deviation reduces from 541 mAh g^−1^ which is industrially not acceptable, to 28 mAh g^−1^. Note the capacities quoted above (814 ± 541 mAh g^−1^ → 1264 ± 28 mAh g^−1^) were all obtained without nanoarchitectured host material, for the benefit of scalable industrial production. That is, we just used raw commercial S_8_ powder without nanostructured hosts, and Ketjen Black as the only conductive additive with no carbon nanotube, graphene, etc. Under these host‐free conditions, TDS gives deplorable performance, while FDS‐C, which can be produced at mass scale, gives excellent and consistent performance.

By avoiding the internal stresses^12^ and volume shrinkage^13^ generated by solvent evaporation in the conventional thermally dried electrodes, high‐sulfur‐loading FDS‐C electrodes of areal loading as high as 14.2 mg cm^−2^ were fabricated and tested in both coin cells and pouch cells, which is the highest sulfur loading based on blade‐casting technique as far as we know. The as‐designed “through‐canals” have low tortuosity from end to end, facilitating Li^+^ transport all the way down to the root (current collector) that contributes to high sulfur utilization even at high sulfur loading; meanwhile, the abundant multiconnected “capillaries” aids lithium polysulfides reaction and retention. Also, the canal/capillary pore structure is robust and can withstand the remodeling caused by the solid S_8_ particle partial dissolution and precipitation during battery operation, as the basic features of multiconnected “capillaries” at 10^1^–10^2^ nm length scale and end‐to‐end canals at ≈10^1^ µm length scale in FDS‐C are still statistically preserved even after long cycles. We discovered that the values of E/S reported in academic literature for coin cells are too high (the lowest value found is >2.8 µL mg^−1^)^14^ to be useful in guiding the construction of pouch cells. Indeed, quantitative calculations show that some high‐performance Li–S pouch cells touted by industry (full‐cell energy density > 400 Wh kg^−1^) are unlikely to be actually guided by the E/S set by academic literature for coin cells. In this paper, we will show that AE/S, rather than E/S, is the more transferrable measure from coin cells (≈10^1^ mAh capacity) to pouch cells (≈10^3^ mAh capacity and above), as AE, together with the FF, are cathode microstructure‐intrinsic, while E is also influenced by cathode‐extrinsic cell factors (headspace volume, wetting with containers). We will show that amazingly, both coin cell and pouch cell can still work at an (AE/S)_min_ = 1.2–1.3 µL mg^−1^, much lower than what the academic literature reported before for ether‐based electrolytes and S_8_‐polysulfide chemistry. As we sweep AE/S from 2 to 1.2 µL mg^−1^, the electrode pores went from fully flooded (fill factor = 1) to semi‐dry/flooded (FF → 0.6), and the coin cell and pouch cell can still maintain function until (AE/S)_min_ near 1 µL mg^−1^ is reached. Under such an ultralean electrolyte condition the practical energy density of Li–S pouch cell can reach 481 Wh kg^−1^, using commercial S_8_ powders without any esoteric nanoarchitecturing, Ketjen Black as the only conductive additive (no carbon nanotube, graphene, etc.), and Al foil current collector.

## 2 Results and Discussion

As a standard technique used in fabricating porous ceramics^15^, ^19^ and carbonaceous materials,^16^ lyophilization (also called freeze‐drying) has been widely adopted in the preparation of hierarchically porous materials. In particular, highly aligned micropores with interconnected nanopores were generally obtained with lyophilization.^19^ In addition, lyophilization can eliminate internal stress generated during the solvent evaporation, and therefore, is beneficial for manufacturing thick electrodes without cracks that were usually generated in the thermal‐drying process because of the huge volume shrinkage.^13^, ^17^ The electrode fabrication process by freeze‐drying is schematically shown in Figure S2a in the Supporting Information. Briefly, a uniform slurry composed of raw commercial sulfur powder, KB and LA133 binder was cast on Al current collector and then lyophilized at −40 °C. Considering the high initial total porosity (P) of freeze‐dried sulfur (FDS) electrode, which demands large amount of electrolyte to fill, we further compressed the as‐obtained sulfur cathode (defined as FDS‐C). For comparison, we also prepare the electrode by conventional thermal drying method (TDS electrode) using the same blade‐casting slurry.

The optical picture in **Figure** **2**a reveals a macrocracked morphology of TDS at 10^3^ µm length scale on the electrode surface, due to the huge surface tension forces and volume shrinkage during the evaporation process of electrode slurry solvent, as illustrated in Figure S4a in the Supporting Information. These electrode macrocracks are detrimental because they would act as “lakes” reserving a large amount of electrolyte that barely serves purposes (a) and (b), and just resulting in higher E/S ratio. From the cross‐sectional scanning electron microscope (SEM) image in Figure 2b, two consecutive layers (divided by the cyan dotted line) were observed, with an upper dense layer and a porous layer beneath. The surface SEM in Figure 2c confirms a compact polymer‐enriched morphology that impedes electrolyte imbibition. The magnified surface morphology inset of Figure 2c indicates that the particles are packed tightly together due to the localization of polymer binder. The upper dense layer is hard for the electrolyte to penetrate, sealing off pores in the bottom layer. On account of only one binder polymer existed in the electrode slurry, we conclude that the upper dense layer is caused by the binder condensation during the solvent evaporation and subsequent cooling‐off process. From the magnified image of the dense layer in Figure S4c in the Supporting Information, it is also visually observed that a lot of binders aggregated in the upper layer as marked by the yellow dotted circle. Semiquantitative elemental analysis by energy dispersive spectrometer indicated binder accumulation on the electrode surface as the ratio of sulfur in the binder‐enriched area decrease, shown in Figure S4e in the Supporting Information. Polymer binder localization is a common phenomenon in film preparation during the thermal drying process, in ceramic films^18^ and LIB electrode preparation.^11^ The evaporation of solvent from the inside of the film to the surface inevitably disturbs the originally uniform binder distribution and enrich it on the surface, as schematically shown in Figure S4a in the Supporting Information. However, this phenomenon is less well known in the Li–S battery community, which should have been paid more attention to, for the dense upper layer may seal off the pores beneath and hinder the electrolyte penetration, harming both electrolyte functions (a) and (b) (especially function (b)). Also, as revealed in Figure 2b, the pore structure beneath shows an irregular distribution due to stresses.

**Figure Figure 2:**
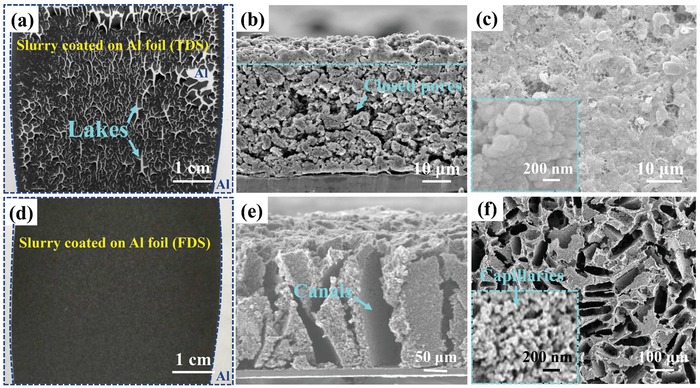
The comparison of the pore structures between traditional thermally dried sulfur (TDS) electrode and freeze‐dried sulfur (FDS) electrode. a) Optical picture of the electrode surface prepared by thermal drying, b) cross‐sectional SEM picture of the electrode prepared by thermal drying, c) surface SEM picture of the electrode prepared by thermal drying and the inset is the magnified surface morphology, d) optical picture of the electrode surface prepared by lyophilization, e) cross‐sectional SEM picture of the electrode prepared by lyophilization, f) surface SEM picture of the electrode prepared by lyophilization, and the inset is the magnified surface morphology in FDS‐C. The sulfur loading of all the cathode in this experiment was controlled at 4 mg cm^−2^.

In contrast, as revealed in Figure 2d, FDS prepared by lyophilization shows a macrocrack‐free surface morphology due to the support of surface tension force by ice crystals in the drying process,^19^ which enables one to fabricate high‐loading damage‐free electrode. Figure S3 in the Supporting Information shows that the sulfur electrode remains macrocrack‐free even at a sulfur loading as high as 14.2 mg cm^−2^. From the surface and cross‐sectional SEM images of the as‐prepared sulfur electrode (at a sulfur loading of 4 mg cm^−2^) by lyophilization in Figure 2e,f, a lot of aligned channels with 227 µm in depth and ≈50 µm in width are observed within the cathode. The formation process is illustrated in Figure S2b in the Supporting Information, where the vertical pores are left by sublimation of the ice dendrites template. Note that those aligned pores have low tortuosity all the way down to the current collector, robust enough to resist clogging or structural changes caused by the solid dissolution and reprecipitation, and therefore can work as “highways” to transport Li^+^ efficiently end‐to‐end, fulfilling function (a) of electrolyte.^20^ However, sulfur cathodes with such abundant through‐canals would undeniably be deficient in compaction density and volumetric energy density. Therefore, we further calendered the as‐obtained FDS electrode. Surprisingly, even after compression by a factor of ≈2, these “through‐canals” are well maintained only with some shrinkage in size, as shown in Figure S4f in the Supporting Information, and the electrode thickness after compression is 103 µm, giving a normalized electrode thickness of ≈25.7 µm per 1 mg cm^−2^ S_8_, which is impressive compared to the previous report.3e Meanwhile, Figure S4g in the Supporting Information reveals the electrode surface morphology after compression, indicating the width of “through‐canals” has been reduced to ≈10 µm, as the through‐canals offer larger free volume for compaction. From the enlarged SEM image of the FDS surface in the inset of Figure 2f, a well‐distributed multiconnective hierarchical pore structure has been achieved, thanks to the surface tension‐force‐balance in forming FDS. These multiconnected “capillaries” with 10^1^–10^2^ nm length scale are suitable to facilitate LiPS dissolution and reaction (electrolyte function (b)) at the smallest size. As reported by Chen et al.^21^ and Fang et al,^22^ these capillaries are beneficial for confining LiPS species, which means that capillary not only promotes the LSM but also suppresses GSM. In addition, the surface tension‐force‐negation fabrication process also contributes to a homogeneous pore (both canal and capillary) distribution in the electrode, which would be crucial to attaining a stable sulfur utilization, as we will discuss later.

From the pore size distribution in **Figure** **3**a, a pore size around 50 µm was found in the FDS which correlates well with “canals” as shown in the SEM pictures in Figure 2e,f. Besides, a weak broad peak from 10^2^–10^3^ nm that represents the capillary pores is also found. Even after compression (FDS‐C), FDS kept a well‐preserved hierarchical canal/capillary microstructure, which shows ≈10 µm width “canals” and ≈10^2^ nm multiconnected “capillaries,” respectively, corresponding well to the SEM images in Figure S4f,g in the Supporting Information. For comparison, the pore size distribution of the TDS shows no obvious peaks at either micrometer or nanometer length scale even though a lot of irregular pores were found from the cross‐sectional SEM image in Figure 2b. Thus, we conclude that the pores within the TDS electrode are mostly sealed off by the binder enrichment layer and exist as closed pores. In addition, in Figure S5 in the Supporting Information, N_2_ adsorption–desorption test also reveals that the FDS‐C has more “capillary” pores than TDS in spite of compression. One may call the pore size distribution in FDS‐C “bimodal,” although the word bimodal does not delineate other key features like pore connectivity, percolation, and tortuosity. So we still prefer the “canal/capillary” descriptor.

**Figure Figure 3:**
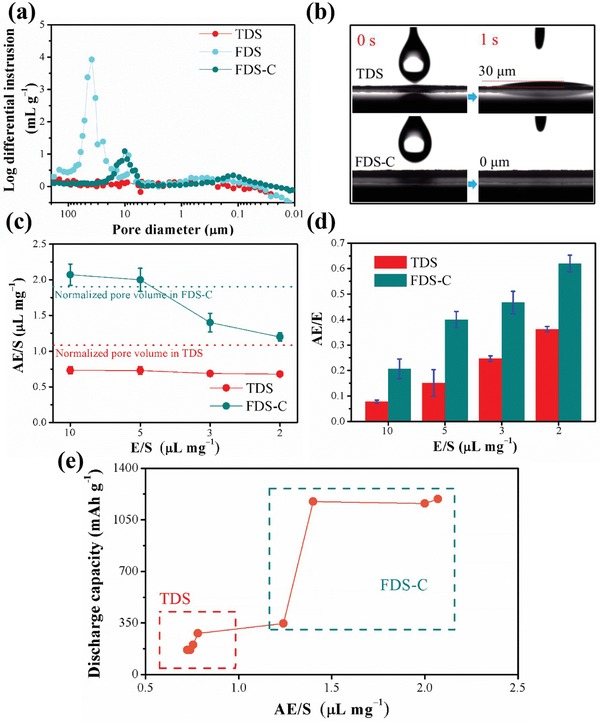
The quantitative pore size distribution, electrolyte permeability, and electrolyte absorptivity study of different electrodes. a) Pore size distribution of different electrodes examined by mercury intrusion porosimetry, b) contact angle between electrolyte and electrode after 5 µL electrolyte was dropped on the electrode surface, c) comparison of the normalized absorbed electrolyte (AE) per 1 mg cm^−2^ S_8_ between TDS and FDS‐C under different E/S ratio, d) comparison of the AE/E between TDS and FDS‐C under different E/S ratio, and the higher AE/E ratio in FDS‐C means that FDS‐C is more tolerant to work under lean electrolyte condition because more AE can be used to sustain functions. e) The relationship between Li–S battery discharge specific capacity and AE/S ratio in coin cells.

To verify the advantage of the canal/capillary structure on electrolyte absorptivity, 5 µL electrolyte (E) was dropped onto a 12 mm electrode disk. As shown in Figure 3b, for TDS, a residual microdroplet (RE) of 30 µm in height was observed on the electrode surface and cannot permeate into the electrode after 30 s (see in Figure S6, Supporting Information); in striking contrast, the same amount of electrolyte is completely absorbed by FDS‐C immediately. The excellent electrolyte permeability of FDS‐C, which is attributed to the open pores, including both the “through canals” of low tortuosity and the multiconnected “capillaries,” facilitates electrolyte absorption under the same E/S ratio.

Specifically, we have quantitively evaluated the electrolyte absorptivity (AE/E) through measuring the AE in the electrodes under different E/S ratios. The AE of the electrodes were measured according to AE = *M* − *m*, where *m* and *M* are the cathode weight before and after long‐term exposure to E. In order to reflect the real electrolyte infiltration condition of the electrodes in coin cell, the infiltration process was conducted by assembling coin cells with different E/S ratio and then rested at room temperature for 2 h. After that, the coin cells were transferred to −20 °C for 30 min before disassembling (in order to reduce the volatilization of the electrolyte during the weighting process). The value of *M* was measured by disassembling the coin cells and weighing the cathode discs, after wiping off the residual liquid by a tissue paper. The AE/E was dimensionless and the value is from 0 to 1, while the unit of AE/S is µL mg^−1^. Compared to FDS‐C, we find TDS tends to have a low AE/E ratio, likely due to binder evaporation‐and‐sealing from the high‐temperature solvent evaporation and cooling‐off process.^11^ So even under a superabundant electrolyte condition like E/S = 10 µL mg^−1^, the inside of TDS can still be *depleted of liquid electrolyte*. Such situation is reflected by the AE/S results in Figure 3d, where TDS has lower AE/S than FDS‐C at all E/S ratios, even lower than the normalized pore volume, indicating there is a large amount of closed‐off pores (the fraction of CP is estimated to be: 1–0.73/1.12 = 34%, where 0.73 is the value of AE/S at E/S = 10 µL mg^−1^, 1.12 is the normalized pore volume *P* in TDS and the detailed calculation can be found in Note S2, Supporting Information). This definitely would disrupt cathode function (a) and (b), leading to poor average performance and very scattered cell‐to‐cell performances, i.e., poor performance consistency, characterized by a large standard deviation in the measured cell discharge capacity from cell to cell, as we will show later. On the contrary, with our newly prepared freeze‐dried‐and‐compressed sulfur cathode, one can eliminate most of the “bad” pores, leaving only the “good” canals and capillaries, so that the electrode pores are fully flooded (fill factor = 1) when adequate electrolyte is supplied (high E/S ratio). Note that the AE/S ratio at E/S = 10 and 5 µL mg^−1^ corresponds well with the normalized pore volume (*P*) in FDS‐C (the green line in Figure 3c) calculated by the difference between measured electrode volume and ideal densities (Note S2, Supporting Information), which verified that the AE/S values measured in our experiment are reliable. Moreover, for FDS‐C electrode, we find when we sweep AE/S from 2 to 1.2 µL mg^−1^, the electrode pores went from fully flooded to semi‐flooded (fill factor → 0.6). Thus, taking analogy with real‐world canals again, the riverbeds are partially exposed in dry season. But encouragely, from the voltage‐capacity curves in Figure S7 in the Supporting Information, it appears that high sulfur utilization could still be achieved even if the electrode is semi‐dry/flooded by ether‐based electrolytes. However, once AE/S drops to ≈1.2 µL mg^−1^ (corresponding to E/S = 2 µL mg^−1^) in coin cells, the FDS‐C fails to discharge/charge properly (the coin cells were tested at room temperature, 25 °C), and therefore, we speculate the lower bound of AE/S estimated from coin cells is around 1.2 µL mg^−1^, namely (AE/S)_min_ ≈1.2 µL mg^−1^. Figure 3e summarizes the relationship between the discharge specific capacity (sulfur utilization) of Li–S battery and AE/S ratio, which reveals the performance is closely related to AE/S instead of E/S ratio. Therefore, it is reasonable to conclude that rather than E/S, which is sensitive to cell size, geometry and packaging material and thus usually not transferrable from coin‐cell to pouch cell, AE/S is the intrinsic parameter that determines the performance of Li–S battery.

One big challenge when scaling up Li–S batteries from the lab‐ to industry‐scale is performance consistency.^23^ While a common practice in academic literature is to assemble several cells and report the *best* cell data, this practice is obviously not acceptable in industry. Herein, other than the routine electrochemical characterizations, we also pay close attention to the variation of Li–S cell performance, which is evaluated by the standard deviation of battery performance of the same batch of cells, made by nominally the same materials and process. It can be seen from **Figure** **4**a that Li–S batteries with TDS exhibit both poor electrochemical consistency and poor average sulfur utilization even at a low sulfur loading of 1.5 mg cm^−2^ and superabundant electrolyte condition (E/S = 10 µL mg^−1^). As shown in Figure 4c, in four nominally identical TDS batteries, the average discharge capacity is only 814 mAh g^−1^ and the standard deviation reached 541 mAh g^−1^. Such wild fluctuations would be unacceptable for industry. This kind of poor performance consistency is typical of what one gets with raw “host‐less” commercial S_8_ powder, Ketjen Black as the only conductive additive (no carbon nanotube, graphene, etc.), and 2D foil current collector. By nanostructuring S_8_ with other nanomaterials before mixing with Ketjen Black, one can get better and more consistent performance, but at the expense of time, cost, and scalability. On the contrary, with the same host‐less sulfur, the charge/discharge curves of FDS‐C electrode exhibit unprecedented consistency and excellent sulfur utilization. The average discharge capacity reached 1264 mAh g^−1^, 55% higher than that of TDS; but even more importantly, the standard deviation among four cells is only 28 mAh g^−1^, nearly 20‐fold smaller than that of TDS, as shown in Figure 4b,c. This proves that the pore microstructure and electrolyte morphology play a crucial role in S_8_ electrode performance, which is one of the central theses of this paper.

**Figure Figure 4:**
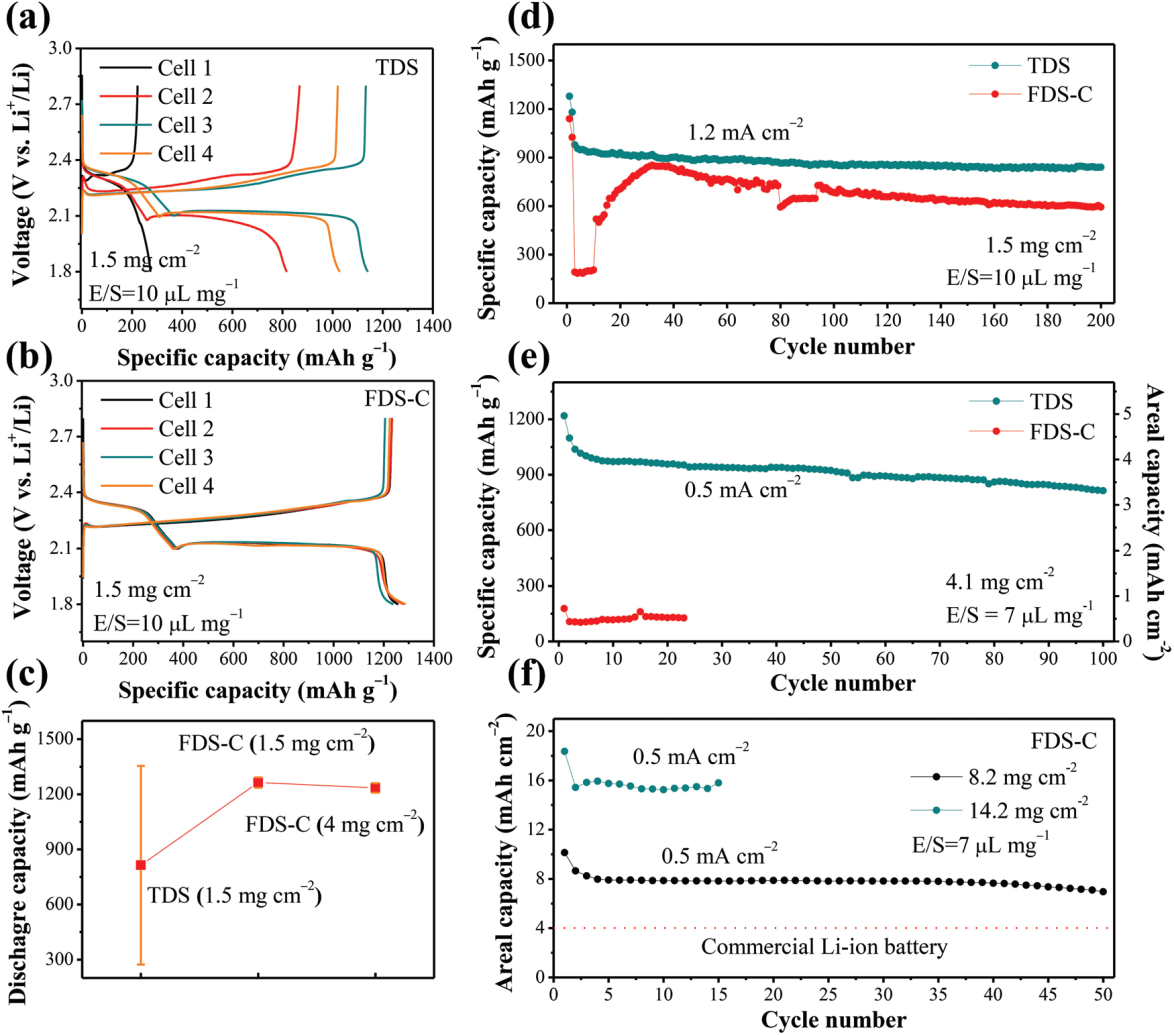
Electrochemical performance of Li–S battery with the TDS and compressed FDS (FDS‐C). The sulfur utilization and cell‐to‐cell consistency study of Li–S batteries with a) TDS and b) FDS‐C, respectively, each sample was tested at four parallel cells. c) The comparison of the average discharge capacity of Li–S batteries with different electrodes and sulfur loading, the error bars in the picture represent the standard deviation of four parallel cells. (d) and (e) are the comparison of the cycling performance between TDS and FDS‐C with different sulfur loading and E/S ratio. f) Cycling performance of Li–S battery with FDS‐C at high sulfur loading.

In addition to the superior capacity and consistency, cycling performance of Li–S batteries with FDS‐C is also much improved. From Figure 4d, FDS‐C batteries demonstrate high capacity retention of 86% after 200 cycles and have a similar capacity fading rate in four nominally identical batteries (Figure S8c, Supporting Information). However, for TDS batteries, the cells display poor cycling stability and high variation (Figure S8b, Supporting Information). The poor sulfur utilization and consistency in TDS indicate that even under superabundant external electrolyte condition (E/S as high as 10 µL mg^−1^), the inside of TDS is still *depleted of liquid electrolyte* (AE/S less than 1 µL mg^−1^) that made it impossible to serve its basic functions. In contrast, the higher AE in FDS‐C serves the two functions well and contributes to the much reduced cell‐to‐cell performance variation. Also, the superior cycling stability indicates that the multiconnected “capillary” not only enhances LSM to guarantee the LiPS dissolution and reaction but also cutting down GSM due to the better adsorption of LiPS. At a sulfur loading of 4 mg cm^−2^, FDS‐C can still deliver a capacity beyond 1200 mAh g^−1^ at the initial cycle while maintaining excellent consistency (Figure S8a, Supporting Information). In addition, the battery also exhibits good capacity retention (as shown in Figure 4e) for 100 cycles. In contrast, Li–S battery with TDS displays exceptionally poor sulfur utilization (less than 200 mAh g^−1^) when the sulfur loading increases to such levels.

The advantage of “through canals” is given a full display in ultrahigh sulfur loading in FDS‐C. Figure 4f shows the cycling performance of Li–S batteries with sulfur loadings of 8.2 and 14.2 mg cm^−2^ at E/S ratio of 7 µL mg^−1^. Notably, the initial high specific capacity of 1250 mAh g^−1^ and a sulfur utilization beyond 75% at such high sulfur loadings indicate that canals are capable of transporting Li^+^ into the thick electrode effectively. With a sulfur loading of 14.2 mg cm^−2^, the areal capacity could reach as high as 18 mAh cm^−2^ in the first cycle and maintain 16 mAh cm^−2^ subsequently, which is ≈5 × that of the commercial lithium‐ion battery cathode. It is worth mentioning again that our super‐thick electrode results above are based on raw commercial “host‐less” sulfur powders without *any nanostructuring*, Ketjen Black as the only conductive additive (no carbon nanotube, graphene, etc.), and 2D current collectors, all of which have been considered inapplicable in the thick electrode before.^13^, ^17^ Pore microstructural engineering thus gave an extraordinary electrochemical performance in FDS‐C, which is also supported by the postmortem examination of FDS‐C electrode that cycled 50 times. From the cross‐section and surface SEM images in Figure S9 in the Supporting Information, we can identify wide canals (10^1^ µm width) in FDS‐C that percolate directly from bottom to the top, which, even though not as straight as that in FDS, are effective for long‐range transport. The pore size distribution in Figure S10 in the Supporting Information further confirms the existence of open pores with ≈10 µm width within the electrode, indicating the robust nature of the “through canals” during cycling. We cannot identify capillaries by high‐resolution SEM observation (Figure S9d, Supporting Information), mercury intrusion porosimetry (Figure S10, Supporting Information), or N_2_ adsorption–desorption analysis (Figure S11, Supporting Information) of cycled FDS‐C electrode, likely due to choking‐up with residual electrolyte salts once the solvent evaporates. Even after soaking in 1,2‐dimethoxyethane (DME) overnight, it is still difficult to diffuse away the salt, which proves the excellent electrolyte retention capability of capillary pores. With such a combination of robust canals and electrolyte‐reserving capillaries, stable cycling is supported.

To further illustrate the effects of pore microstructural engineering, we conduct in situ electrochemical impedance spectroscopy (EIS) on both TDS and FDS‐C electrodes. EIS tests were conducted every 2 h during the discharge process (marked by the stars in **Figure** **5**a), and before the test, the battery was rested for 5 min. For TDS, two typical characteristics, including large polarization at the end of the first platform and poor Li_2_S nucleation kinetics during the second platform, that are common behavior of Li–S chemistry operating under lean electrolyte condition, could be observed in Figure 5a,^14^, ^24^ and the polarization voltage is 4× larger than that of FDS‐C. From the EIS curves shown in Figure 5b, we can see a striking difference in the interphase contact resistance (*R*_int_, the high‐frequency semicircle) and charge‐transfer resistance (*R*_ct_, the medium‐frequency semicircle)^25^ during the discharging process between TDS and FDS‐C. Further EIS curve fitting was done in Figure 5c, and all of the resistances (*R*_ct_, *R*_int_, and electrolyte resistance *R*_S_) exhibit a rise during the first platform, reaching a maximum at the end of the first platform, which may be aroused by the increasing polysulfides concentration in the liquid electrolyte that reduced the Li^+^ conductivity (*R*_S_) and electrochemical reaction kinetics (*R*_int_ and *R*_ct_).^26^ However, the resistance growth rate reveals some discrepancy for TDS and FDS‐C, especially in the *R*_ct_ and the *R*_int_: the *R*_ct_ of TDS increased from 10.5 to 187.1 Ω at the end of the first platform; as a comparison, the *R*_ct_ of FDS‐C increased from 4.9 to 31.4 Ω. It is generally believed that the *R*_ct_ is closely associated with the polysulfides concentration and reflected the nucleation process of Li_2_S/Li_2_S_2_.^24^ Therefore, we speculate that the low AE within TDS will increase *R*_ct_ due to the limited electrolyte used to serve function (b). Besides, growth in the *R*_int_ of TDS (from 16 to 50 Ω) also demonstrates a much more rapid degradation than FDS‐C (from 27 to 34 Ω). Due to the percolating capillary framework in FDS‐C, the larger surface area of exposed conductive carbon is also beneficial for the Li_2_S/Li_2_S_2_ nucleation uniformly and subsequently retards the electrode passivation.^21^ As the discharge continues, all the resistance decreased owing to the consumption of the solubilized polysulfides.^26^ However, due to the serious surface passivation, *R*_int_ in TDS reduces slower as compared to the others. The poor Li_2_S deposition kinetics in the second platform is bad for delivering capacity in Li–S battery as we can see in Figure 5a. Because of the large internal resistance, the voltage of the second platform that comprises ≈75% of the capacity, drops below 1.8 V, and therefore one would lose this capacity in TDS if the discharge voltage was cut off at 1.8 V.

**Figure Figure 5:**
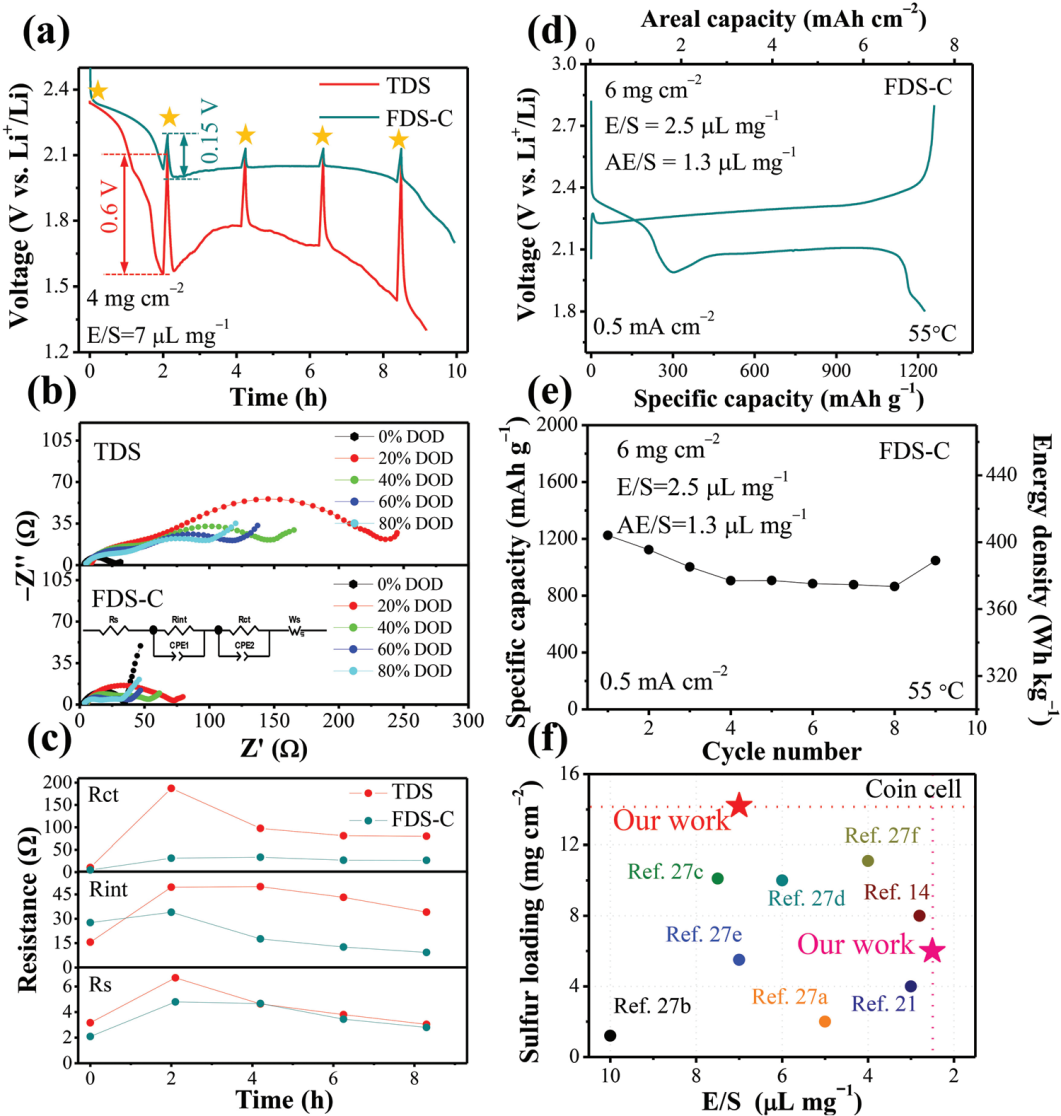
EIS study of Li–S battery with different electrodes and the electrochemical performance of Li–S battery under low E/S ratio. a) The discharge curves of Li–S battery with TDS and FDS‐C, respectively, the measurement interval was 2 h, and before each EIS test, another 5 min was needed for rest. The electrode sulfur loading is 4 mg cm^−2^, and the E/S ratio is 7 µL mg^−1^. b) the EIS curves of Li–S battery at different depth of discharge and the inset is the fitting model used to calculate the impendence kinetics data, c) impendence kinetics data plotted as a function of discharge depth with different electrodes, d) discharge and charge curves of Li–S battery at 2.5 µL mg^−1^ and 55 °C, e) cycling performance of Li–S battery under 6 mg cm^−2^ and E/S = 2.5 µL mg^−1^, AE/S = 1.3 µL mg^−1^, the energy density was calculated with an infinity capacity Li–S pouch cell model and the package materials can be ignored, f) the comparison of the sulfur loading (2D current collector) and E/S ratio of the Li–S battery in recent works on lean electrolytes.

In addition, we also find that the Li–S battery with FDS‐C works well with 1225 mAh g^−1^ reversible capacity and an areal capacity exceeding 7 mAh cm^−2^, even at an ultralow E/S ratio of 2.5 µL mg^−1^, as shown in Figure 5d,e, which is also the lowest E/S ratio reported in ether‐based electrolytes. Furthermore, we measured the AE/S in FDS‐C, which is determined to be 1.3 µL mg^−1^, consistent with the trend in Figure 3d. Considering the normalized pore volume (P) in the electrode is 1.9 µL mg^−1^ (Note S2, Supporting Information), we conclude that the electrode pores must be only *partially filled* by electrolyte, with a fill factor of 1.3/1.9 = 68.4%. Since electrolyte prefers to fill in the narrower pores first due to the capillary attraction, under very lean electrolyte condition, the electrolyte will give priority to function (b), where the “capillary” needs to fulfill to reach high sulfur utilization and low polarization. Drawing analogy to shipping in actual canals during drought period, the “through canal” actually has no need to be fully filled by electrolyte to serve function (a). In such a scenario, we could imagine that the “canals” wall would be wetted, as illustrated in Figure 1a, which still was able to transport lithium ions efficiently due to the percolating pathway. Thanks to the canal/capillary strategy, rational electrolyte partition to sustain both functions (a) and (b) is achieved, guaranteeing an efficient electrolyte usage under ultralean electrolyte condition, which we believe is the key to increasing the gravimetric energy density. Compared with several recent works on lean electrolyte condition of Li–S batteries,^14^, ^21^, ^27^ it can be concluded from Figure 5f that our FDS‐C electrode that used only low‐cost raw material and convenient large‐batch processing method (host‐less) would be highly competitive at lean‐electrolyte and high sulfur loading condition.

Based on the comprehensive understanding above, we next demonstrate the reliability and transferability of our semi‐flooded canal–capillary design *in designing large‐format pouch cells* to enable high‐loading sulfur electrode with lean absorbed electrolyte. **Figure** **6**a,b is digital pictures of the FDS‐C sulfur cathodes that have sulfur loading of 6–8 mg cm^−2^ and the as‐assembled Ah‐level pouch cell, respectively. Three lean and ultralean E/S ratios, 2.3, 1.7, and 1.2 µL mg^−1^, was investigated. Here, for simplicity, the electrolyte stored in separator is neglected because the pore volume in 1 cm^2^ separator is estimated to be 35% (porosity) × 15 µm (thickness) × 1 cm^2^ (area), which is only ≈ 1/30 of that in 1 cm^2^ sulfur electrode at sulfur loading of 8 mg cm^−2^ (70% (porosity) × 216 µm (thickness) × 1 cm^2^ (area)). While in laboratory coin cells the total added electrolyte (E) is not all absorbed inside the cathode considering there is RE in the head spaces and wetting with the cell packaging, when constructing high‐performance pouch cells touted by industry (full‐cell energy density > 400 Wh kg^−1^), it is necessary to cut down RE as much as possible. Take E/S = 2.3 µL mg^−1^ for example, since it has exceeded the nominal pore volume (1.9 µL mg^−1^) in the electrode, there is at least 20% electrolyte as RE. The energy density is only 334 Wh kg^−1^ as shown in Figure S9a in the Supporting Information and Figure 6c. Therefore, in industry such high RE is not allowed. As a matter of fact, in practical Li–S pouch cells that are of large Ah‐level (general > 10 Ah) and as compact as they could be, the headspaces are negligible (RE = 0) and the AE would approximately approach E (AE/E ≈ 1). That is to say, AE/S ≈ E/S in large‐format pouch cells and under lean electrolyte condition. To explore the lower bound of AE/S, we continue to lower down the value to 1.7 µL mg^−1^, which apparently will arrive at a semi‐dry/flooded situation, and the energy density of Li–S pouch cell have already reached 415 Wh kg^−1^, surpassing most of the previous works.

**Figure Figure 6:**
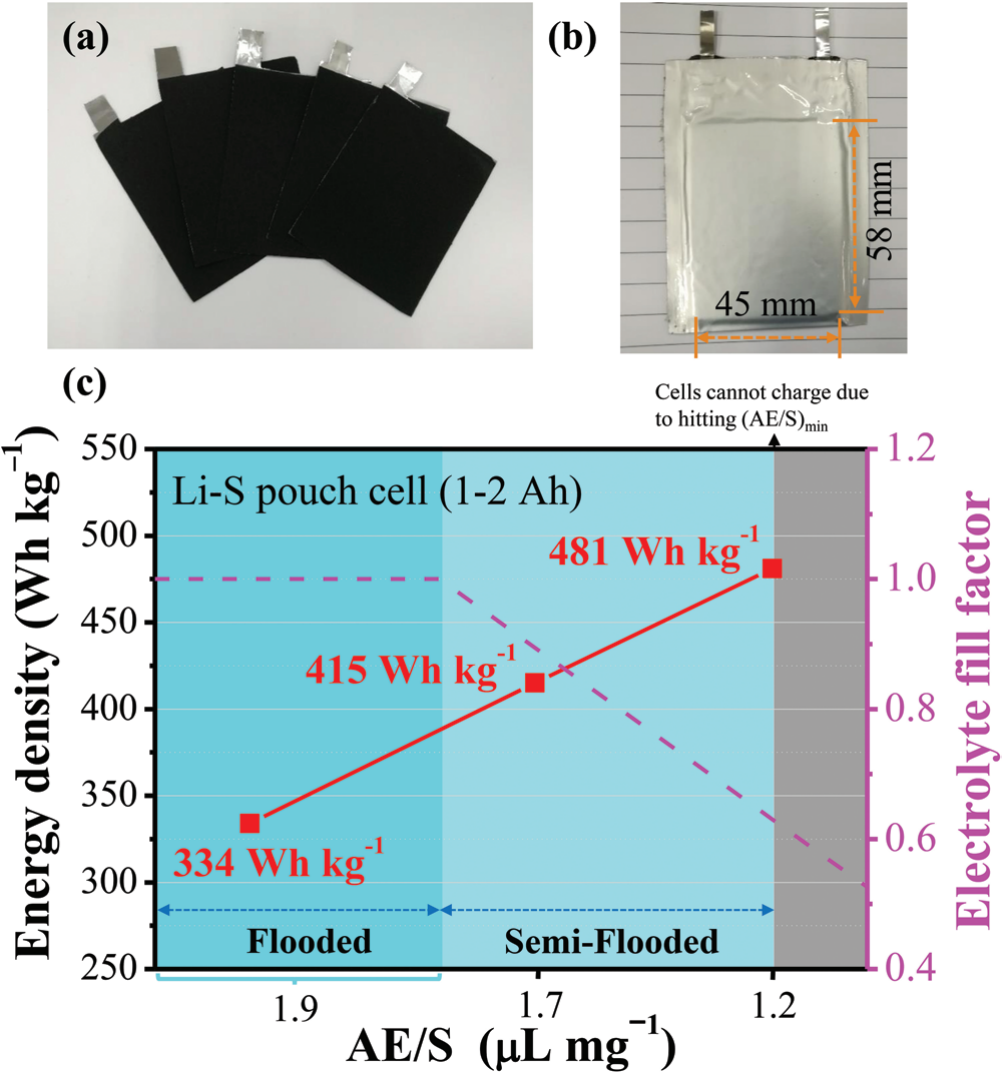
The energy density of Ah level Li–S pouch cell assembled with FDS‐C. a) The picture of sulfur cathodes (FDS‐C) that used to assembling Li–S pouch cell and b) Li–S pouch cell (assembled with three pieces of double side‐coated and two pieces of single side‐coated sulfur electrode). c) The energy density of Li–S pouch cells assembled with FDS‐C under different E/S ratio. The lithium anode used in this experiment is 100 µm (four pieces).

Furthermore, with our Ah‐level pouch cells, when we decrease AE/S to 1.2 µL mg^−1^, a high specific capacity of 1170 mAh g^−1^, namely 70% sulfur utilization, is still maintained in the first discharge, corresponding to an energy density of 481 Wh kg^−1^ in a 1–2 Ah pouch cell. However, after this first discharge, we find our Li–S pouch cells failed to charge normally, even at *T *= 55 °C, which reminds us that 1.2 µL mg^−1^ may be (AE/S)_min_ in Li–S pouch cells with ether electrolyte, below which the battery cannot charge/discharge normally. In other words, we believe that once FF reaches ≈0.6, the canals have dried up so significantly that percolation threshold is breached, and the canals are no longer navigable. Coincidentally, this value (1.2 µL mg^−1^) agrees well with the (AE/S)_min_ in coin cell (as shown in Figure 3e). This measured critical value 1.2 µL mg^−1^ for pouch cell is still much higher than that in LIBs (≈0.3 µL mg^−1^)). One may rationalize it by recalling that with S_8_‐polysulfide chemistry, liquid electrolyte must serve dual functions (a) and (b) in Figure 1, while in LIBs it only needs to serve function (a).

Once again, it turns out that AE/S is the intrinsic parameter that determines the performance of the sulfur cathode. AE/S aligns coin‐cell performance well with practical pouch‐cell performance. In other words, the AE/S value that is measured in coin cell is transferrable to predict the operability of the pouch cell, at a much larger capacity. This finding is very useful for lean electrolyte Li–S battery design because it provides a standard to evaluate the sulfur cathode. In developing new sulfur cathode materials (with or without nanostructured hosts), we do not have to assemble high capacity pouch cell in the early stage, instead, we only need to measure the AE/S in the coin cells to predict the practical pouch cell performance, which is more efficient and economic. With regard to the exact mechanism of FDS‐C failing at/below (AE/S)_min_ ≈1.2 µL mg^−1^, we suspect the drastically increased polarization in the discharge process (Figure S7, Supporting Information) may be aroused by a phase transition of the electrolyte, namely at such low AE/S ratio the solvent molecules may be absorbed by the incompletely dissolved solid Li_2_S*_n_* (the maximum solubility of LiPS in the conventional electrolyte is about 8 M_S_ L^−1^, E/S ratio ≈ 3.9 µL mg^−1^),^28^ turning into a solid phase (or semi‐solid phase) all together (“solvent‐in Li_2_S*_n_*,” analogous to hydrate crystals), in contrast to Li_2_S*_n_* dissolving in solvents when at higher AE/S ratio (“Li_2_S*_n_*‐in‐solvent,” analogous to an aqueous solution). Thus, the “drying of the river bed” may be of a chemical nature, instead of a purely physical or geometric nature.

## 3 Conclusion

To summarize, in this work, we have developed a freeze‐drying approach to fabricate sulfur cathodes with canal/capillary hierarchical pores using raw commercial sulfur powders without nanostructured host, Ketjen Black as the only conductive additive (no carbon nanotube, graphene, etc.), and 2D foil current collectors. Compared with the conventional thermal‐drying electrodes with plenty of macrocracks and closed‐off pores at high sulfur loading, FDS‐C guarantees end‐to‐end highways for lithium ions transport due to templating by subliming ice dendrites and meanwhile lithium polysulfides dissolution and reaction are also facilitated by the nanoscale capillaries, so Li_2_S nucleation barrier is largely reduced even with semi‐flooded electrolyte filling, under very lean electrolyte condition (AE/S = 1.3 µL mg^−1^ in coin cell and AE/S = 1.2 µL mg^−1^ in pouch cell). With pore microstructure engineering, we have achieved unprecedented cathode performance consistency using highly scalable host‐less sulfur. FDS‐C with high sulfur loading and ultralean electrolytes can boost the energy density of Li–S pouch cell to 481 Wh kg^−1^. Our work demonstrates that besides active material design, pore microstructural engineering is the crucial step for enhancing the Li–S battery performance. Also, we have shown that the cathode‐intrinsic quantities evaluated in coin cells, e.g. (AE/S)_min_ ≈ 1.2 µL mg^−1^ and FF < 1, can be directly transferred to pouch cell design and predict its operability.

## 4 Experimental Section

##### Electrode Preparation

All of the electrodes were prepared by a slurry coating procedure. First, commercial sulfur powder (99.8%, Sigma‐Aldrich Chemical Co. Ltd) and KB (Lion Co., ECP‐600JD) were mixed uniformly and heated at 155 °C for 12 h (70 wt% sulfur). After that, the as‐prepared S/KB composites were ball‐milled with aqueous LA‐133 binder (Chengdu Indigo Power Sources Co., Ltd, China) for 6 h at 350 r min^−1^ in a planetary ball milling machine at a mass ratio of 9:1. Thereafter, the slurry was coated onto an aluminum foil current collector at a different thickness. Finally, for thermal‐drying, the as‐prepared electrode was dried at 60 °C for 12 h in a vacuum drying oven. For lyophilization, the as‐prepared electrode was first frozen at −40 °C for 2 h and then vacuumed to 0.1 Pa for 2 h to induce ice crystal sublimation. By using lyophilization the sulfur loading can reach as high as 14.2 mg cm^−2^.

##### Characterization of Electrode

The electrode morphology and microstructures were studied by field‐emission scanning electron microscopy (FESEM, JEOL JSM‐6380LV FE‐SEM). The N_2_ adsorption/desorption tests were fit to Brunauer–Emmett–Teller relationship using an ASAP‐2010 surface area analyzer. The pore size distribution was derived from the desorption branch of the isotherm with the Barrett–Joyner–Halenda method. Besides, the pore size distributions of the electrode were also measured by mercury intrusion porosimetry (Micrometrics, Auto Pore IV 9520, USA).

##### Electrode Electrolyte Infiltration and Absorptivity Test

Electrolyte infiltration measurements were performed by drop casting the droplet of electrolyte and monitoring the wetting process by contact goniometer (Powereach co., Ltd, JC2000C, China). The AE of the electrode was calculated according to AE = *M* − *m*, where *m* and *M* are the cathode weight before and after electrolyte infiltration. In order to simulate the real working condition, the electrode infiltration was evaluated by assembling coin cells with different E/S ratio. The value of *M* was achieved by disassembling the coin cells and then weighing the cathode at different E/S. The cells were rested for 2 h for electrolyte full infiltration before disassembling the battery (the storage temperature is −20 °C). After disassembling the battery, the cathode disc was taken out and the surface was lightly scrubbed by filter paper and then weighed. Each sample was tested at three parallel tests to get the average value.

##### Electrochemical Characterization

CR2025 type coin cells were assembled using different electrodes and Li metal anode in the Ar‐filled glove box. Ah‐level Li–S pouch cells were assembled in dry room with a dew‐point below −45 °C. The electrolyte was 1 m lithium bis(trifluoromethanesulfonyl) imide (LiTFSI) in a 1,3‐dioxolane and DME (2:1, v/v) with 0.6 m LiNO_3_. The total E/S ratio was calculated by the electrolyte volume divided by sulfur mass. The cycling performance of the cells was measured by the galvanostatic charge and discharge within a voltage range of 1.8–2.8 V versus Li^+^/Li at various current densities on Landt 2001A battery cycler. The in situ EIS measurements of the cells were performed using electrochemical impedance spectroscopy (Bio‐Logic, VMP‐300) along with the battery constant‐current discharge. And the measurement interval was 2 h and before each EIS test another 5 min was needed for rest.

Details about the calculation of *E*_g_ are shown in Note S1 in the Supporting Information.

Details about the calculation of electrode pore volume are shown in Note S2 in the Supporting Information.

## Conflict of Interest

The authors declare no conflict of interest.

## Supporting information

Supporting Information
