# Supplementary material for: Semi‐Flooded Sulfur Cathode with Ultralean Absorbed Electrolyte in Li–S Battery
Source: Adv Sci (Weinh). 2020 Mar 18;7(9):1903168. doi: 10.1002/advs.201903168 (PMC7201250; doi:10.1002/advs.201903168)
Supplement: Supplementary file 1 — Supporting Information [file ADVS-7-1903168-s001.pdf]

Copyright WILEY-VCH Verlag GmbH & Co. KGaA, 69469 Weinheim, Germany, 2020.

ADVANCED SCIENCE
Open Access

Supporting Information

for *Adv. Sci.,* DOI: 10.1002/advs.201903168

Semi-Flooded Sulfur Cathode with Ultralean Absorbed Electrolyte in Li–S Battery

Yong Xie, Guoyu Pan, Qiang Jin, Xiaoqun Qi, Tan Wang, Wei Li, Hui Xu, Yuheng Zheng, Sa Li, Long Qie, Yunhui Huang, and Ju Li**

Copyright WILEY-VCH Verlag GmbH & Co. KGaA, 69469 Weinheim, Germany, 2019.

Supporting Information

Semi-flooded Sulfur Cathode with Ultra-Lean Absorbed Electrolyte in Li-S Battery

Yong Xie, Guoyu Pan, Qiang Jin, Xiaoqun Qi, Tan Wang, Wei Li, Hui Xu, Yuheng Zheng, Sa Li, Long Qie, Yunhui Huang, and Ju Li**

Y. Xie, G. Pan, Q. Jin, X. Qi, T. Wang, W. Li, H. Xu, Dr. Y. Zheng, Dr. S. Li, Prof. L. Qie, Prof. Y. Huang
Institute of New Energy for Vehicles, School of Materials Science and Engineering
Tongji University, Shanghai 201804, China
E-mail: lisa@tongji.edu.cn (SL)
Prof. J. Li
Department of Nuclear Science and Engineering and Department of Materials Science and Engineering
Massachusetts Institute of Technology, Cambridge, Massachusetts 02139, USA
E-mail: liju@mit.edu (JL)

Keyword: lithium-sulfur batteries, high-loading electrode, lean electrolyte, canal-capillary microstructure, fill factor

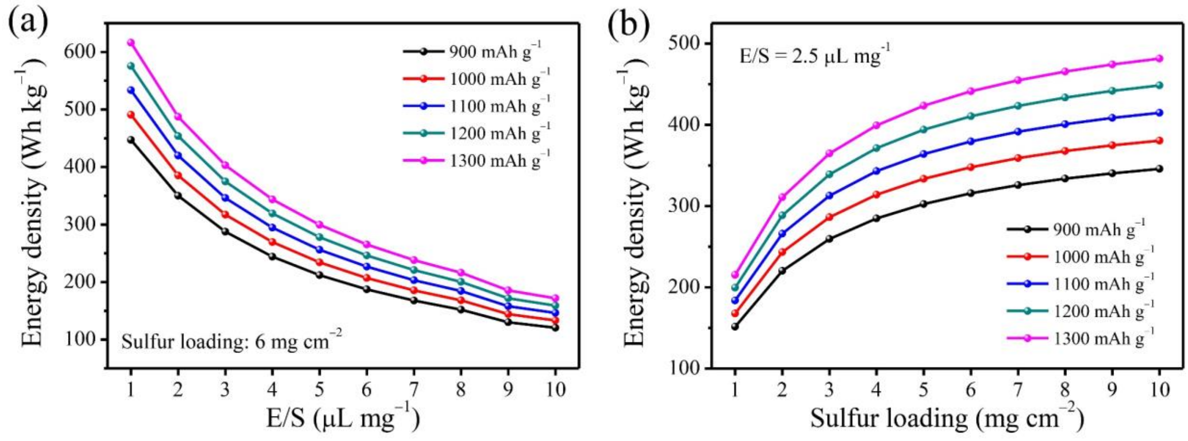

Figure S1. (a) and (b) are the influence of electrolyte/sulfur (E/S) ratio and sulfur loading on Li-S battery energy density respectively. The calculated is based on a formula $E_g = \frac{V \cdot C}{\sum m_i}$, and the mass in denominator including a cathode (sulfur, conductive carbon, binder, aluminum foil), separator, electrolyte, lithium metal anode (2× excess) and copper foil. More detail calculate method can be found in Note S1.

Note S1

The calculation of practical energy density (Wh kg^{-1}) of Li-S battery

$$E_g = \frac{V \cdot C}{\sum m_i}$$

E_g: Gravimetric energy density (Wh kg^{-1});
V: Average cell voltage (V). 2.15 V is assumed for Li-S batteries;
C: Areal capacity (mAh cm^{-2});
m_i: Mass per unit square of various cell components (mg cm^{-2}) including a cathode, an anode

(2× excess of Li for all calculations), Al, Cu current collectors, separator, electrolyte;
Li: 3860 mAh g^{-1}, sulfur content in the cathode: 75%
The thickness of Al: 7.5 μm (slurries coated on both sides), thickness of Cu: 4 μm (slurries coated on both sides), thickness of separator: 25 μm

Table S1 Theoretical densities of components used in Li-S batteries.

Materials	Li	Sulfur	Carbon	Binder	Al	Cu	Separator	Electrolyte
Density ($g\ cm^{-3}$)	0.534	2.07	2.02	1.76	2.7	8.96	0.946	1.2

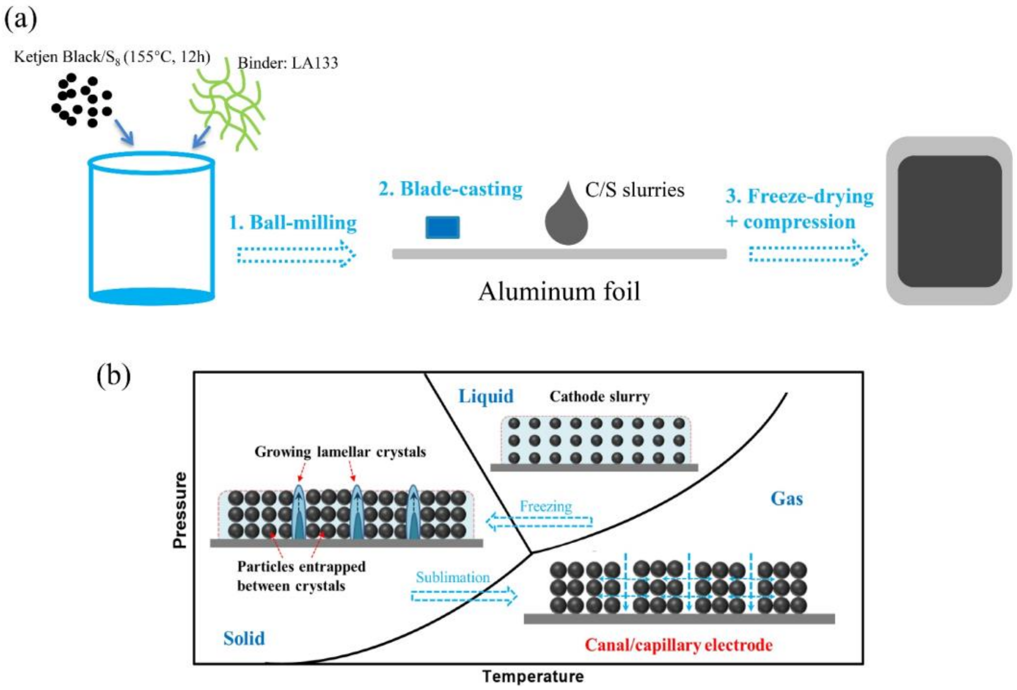

Figure S2 (a) The schematics of the electrode fabrication process through lyophilization, (b) The schematics of physical process of FDS preparation.

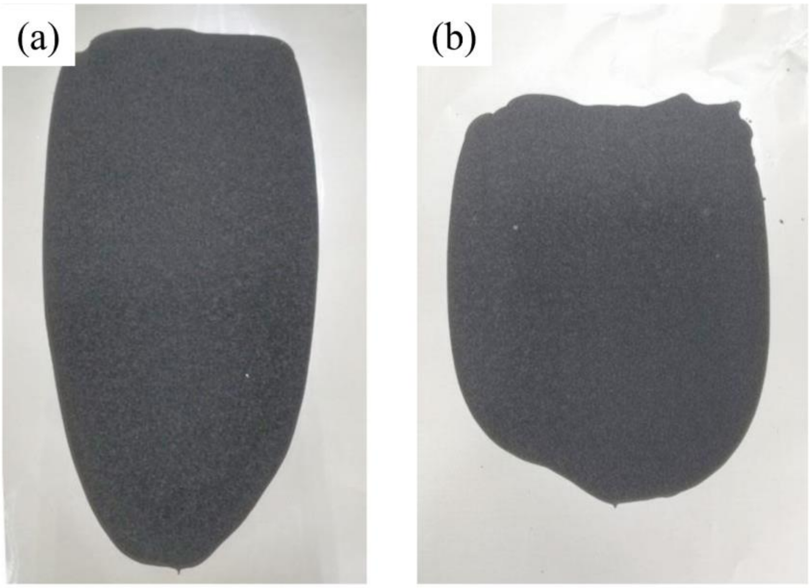

Figure S3. Digital pictures of the electrode surface prepared by lyophilization. The sulfur loading is 10 mg cm^{-2} (a) and 14.2 mg cm^{-2} (b), respectively.

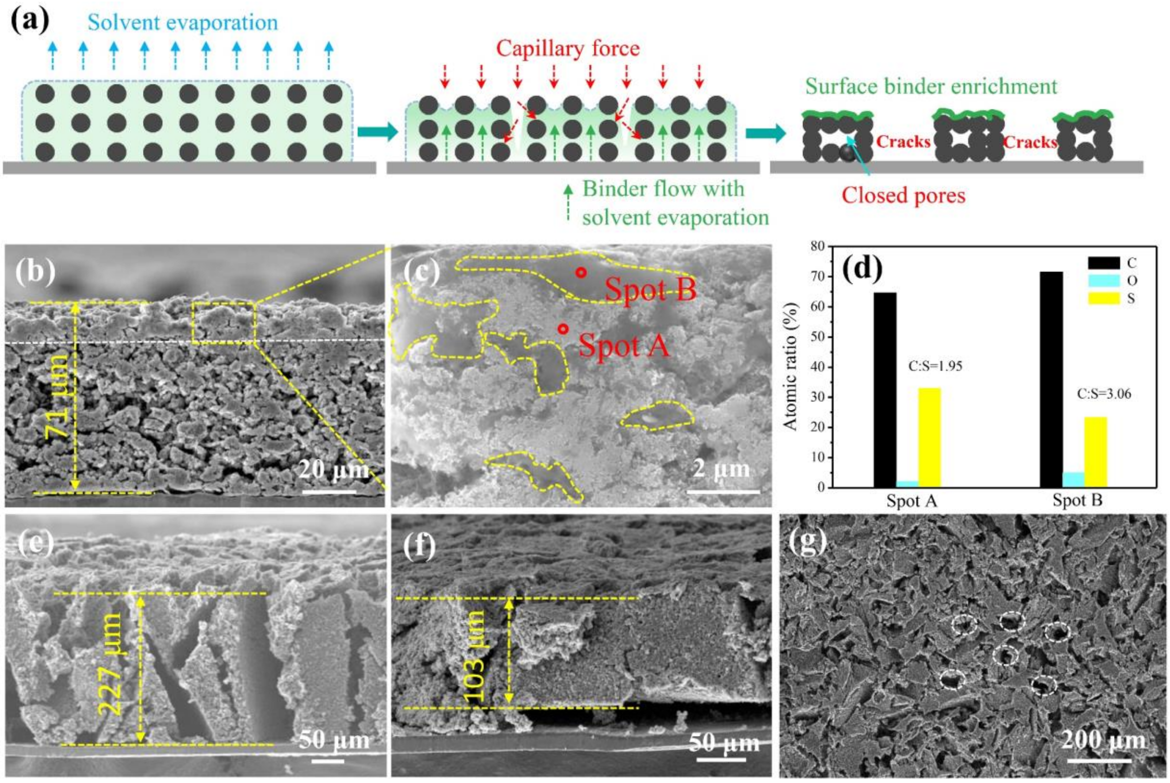

Figure S4. (a) Schematics of the physical process of the electrode prepared by conventional thermal drying (TDS), (b) the cross-sectional SEM of TDS, (c) the magnified picture of the surface layer in (b), the irregular yellow dotted line indicates the binder enrichment area, (d) the EDS element analysis of spot A and spot B, where the high content of carbon in spot B indicates a binder enrichment, (e) and (f) are the cross-sectional SEM prepared by lyophilization before (FDS) and after compressing (FDS-C), respectively, (g) the electrode surface SEM prepared by lyophilization (after compressing). Sulfur loading of all electrodes used in this experiment are 4 mg cm^{-2}.

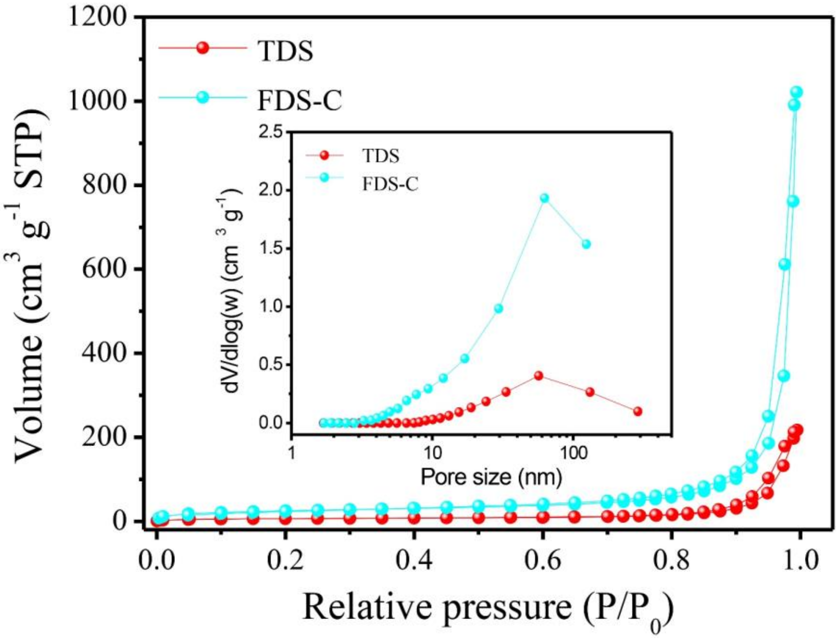

Figure S5. Quantitative electrode pore structure analysis by N_2 adsorption-desorption method. The

surface area of the electrode prepared by thermal-drying and lyophilization are 40.578 m^2 g^{-1} and 114.316 m^2 g^{-1}, respectively.

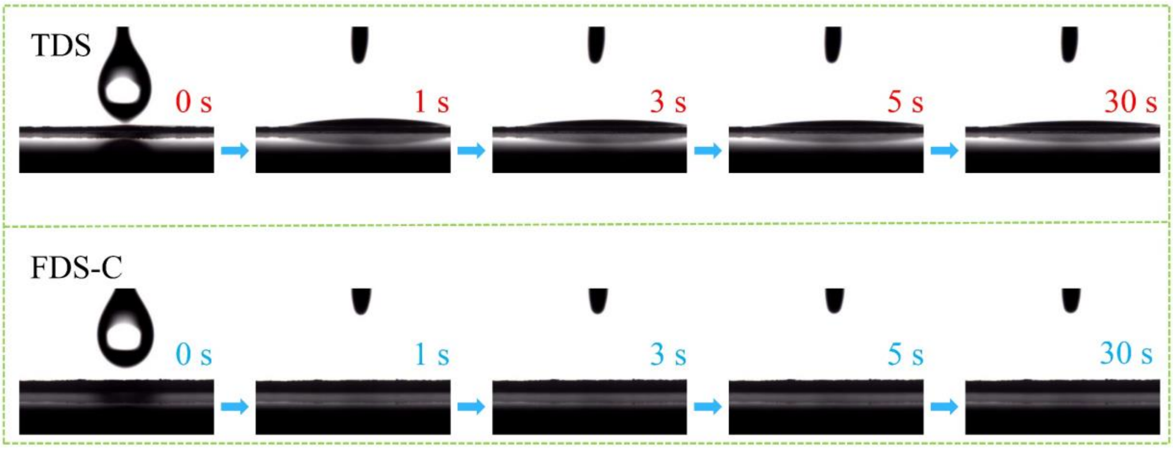

Figure S6. The electrolyte infiltration test of different electrodes.

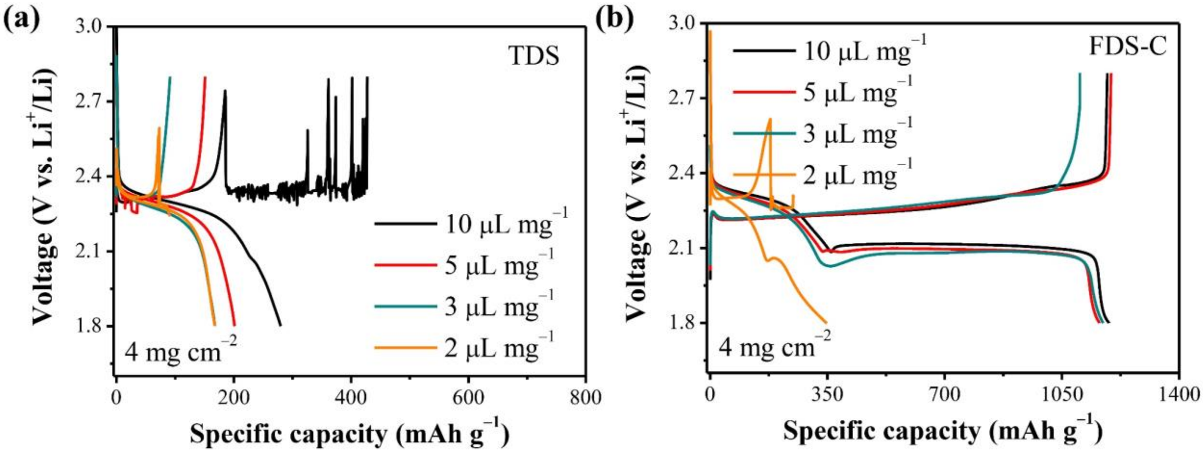

Figure S7. (a) and (b) are the charge/discharge curves of Li-S battery with TDS and FDS-C respectivly at different E/S ratio.

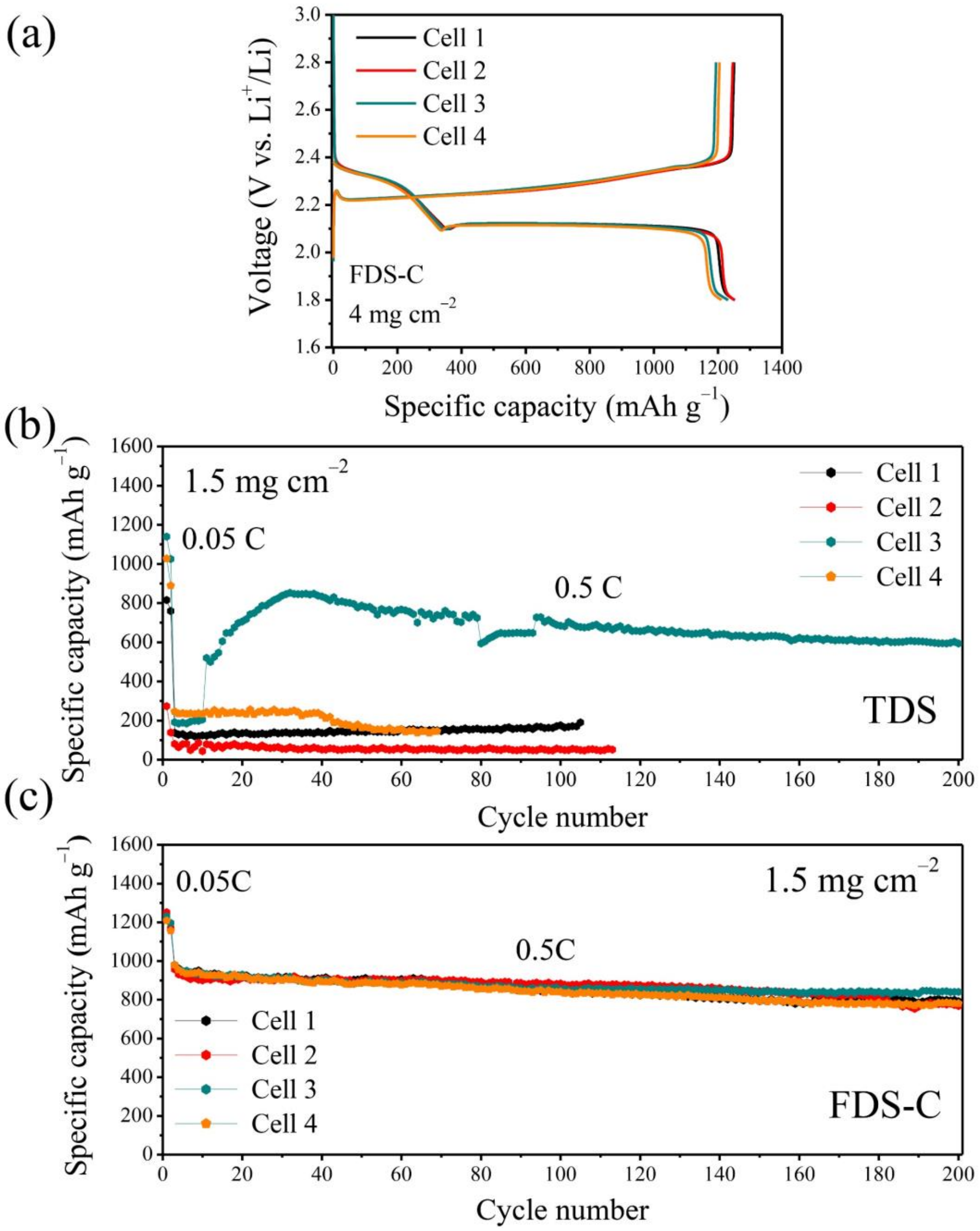

Figure S8. (a) the charge-discharge curves of Li-S battery with a sulfur loading of 4 mg cm^{-2}. (b) and (c) are the cycling performance consistency study of Li-S battery with TDS and FDS-C. All the samples were tested at four identical cells.

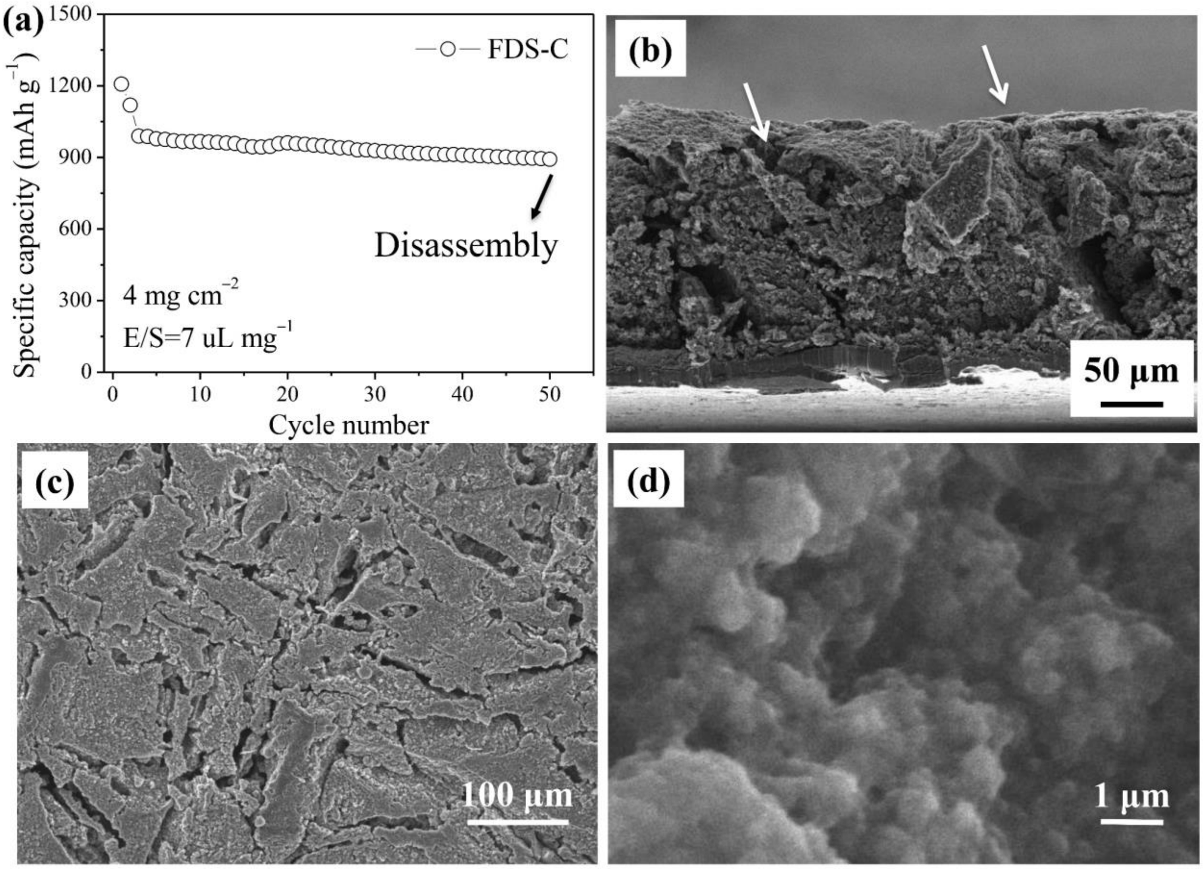

Figure S9. SEM images of sulfur cathode after 50 cycles. (a) Cycling performance of Li-S battery before disassembling, (b) cross-section of FDS-C after 50 cycles, (c) surface morphology of FDS-C after 50 cycles and (d) is the magnifying picture. The battery was disassembled at charge state and the electrode was soaked in DME overnight before the further analysis.

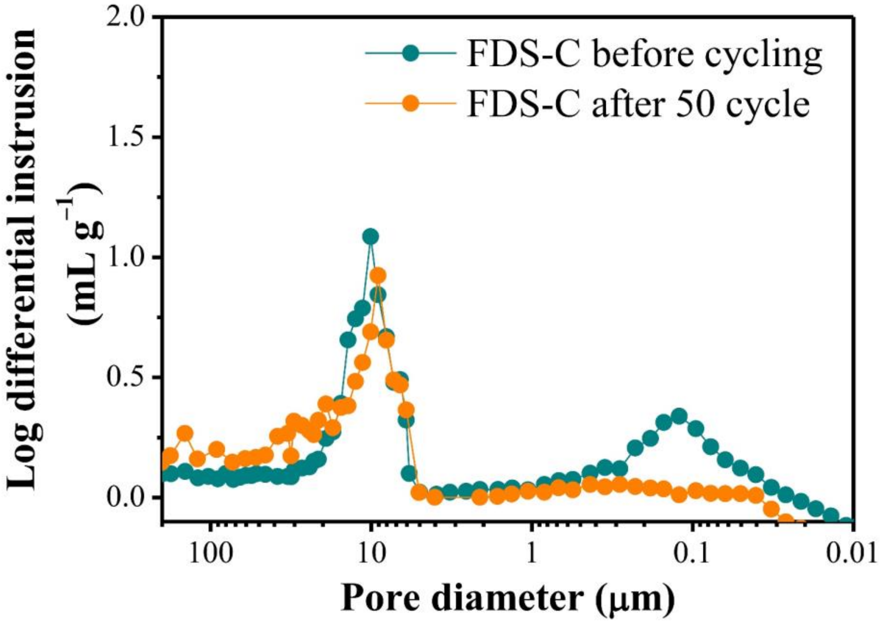

Figure S10. The pore size distribution of FDS-C before and after 50 cycles examined by mercury intrusion porosimetry.

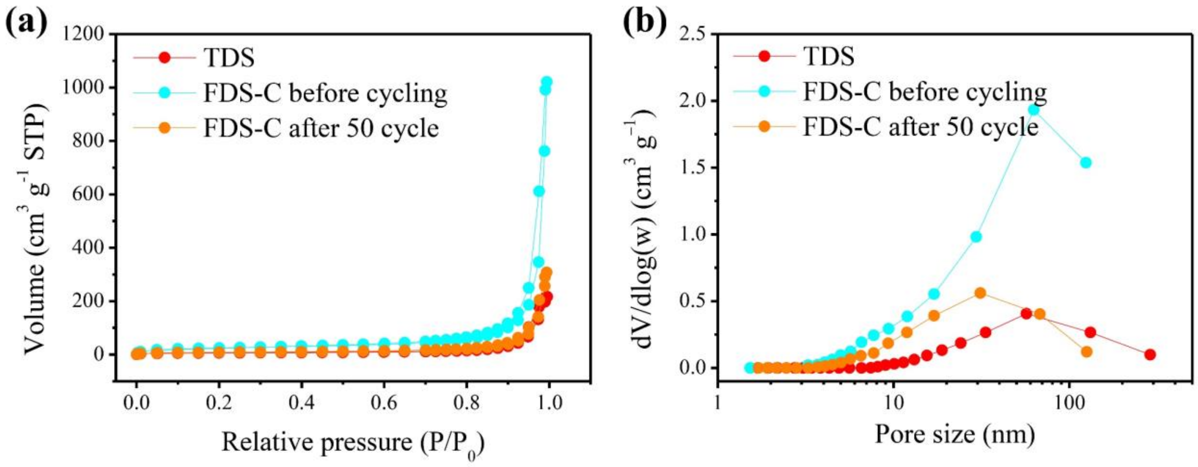

Figure S11. Quantitative electrode microstructure analysis by N_2 adsorption-desorption method.

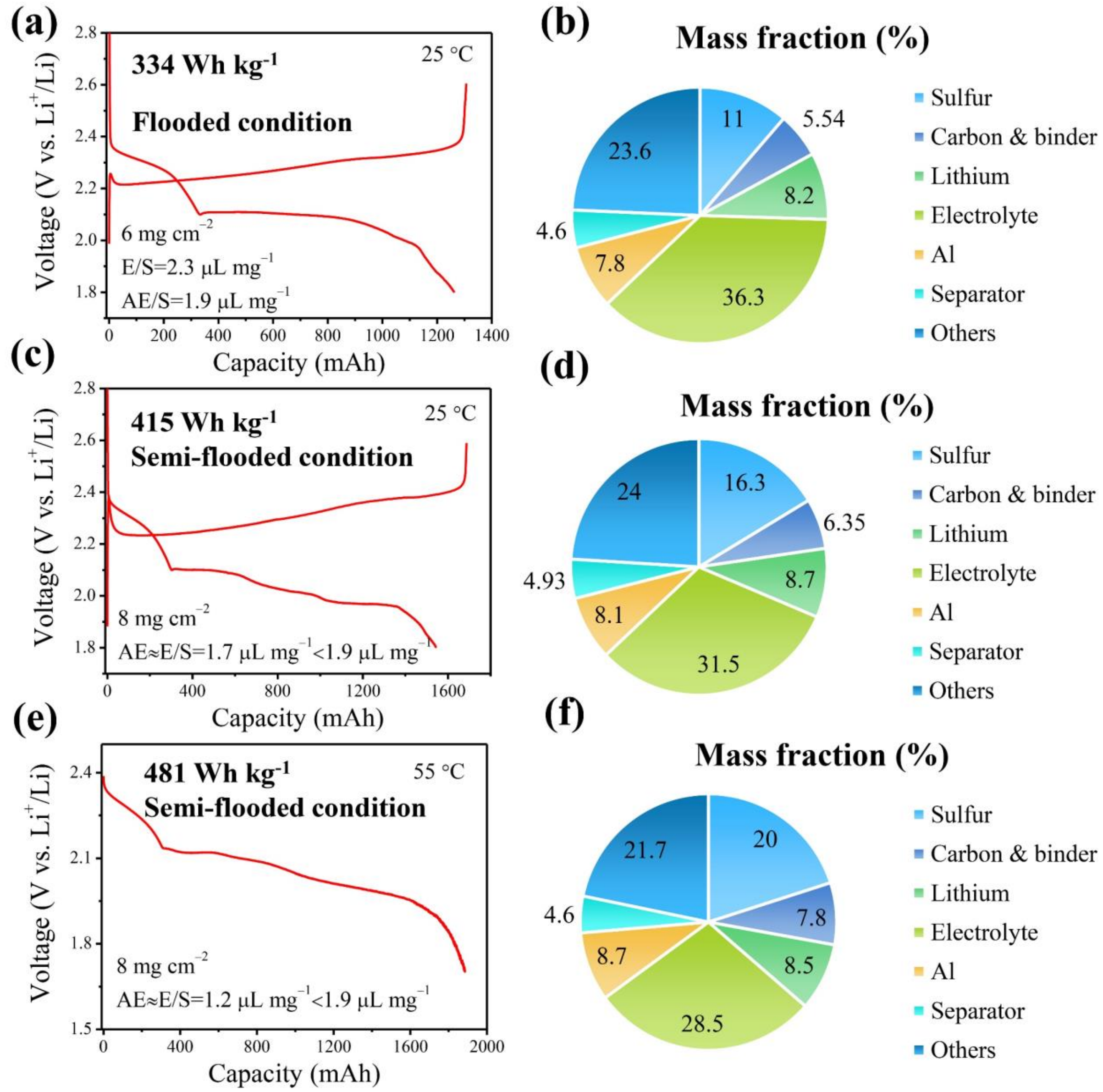

Figure S12. Voltage-capacity curves and mass fraction of Li-S pouch cell at different E/S ratio and sulfur loading. The current density in this experiment is C/30.

Note S2

The calculation of normalized pore volume (V_p) in the electrode

$$V_p = V - (\frac{m_s}{\rho_s} + \frac{m_c}{\rho_c} + \frac{m_{LA133}}{\rho_{LA133}})$$

V: volume of the electrode corresponding to 1 $mg_{(s)}$ cm^{-2}, according to Figure S4, 1$mg_{(s)}$ cm^{-2}~26 μm for FDS-C and 1$mg_{(s)}$ cm^{-2}~18 μm for TDS.

The sulfur is assumed to 1mg cm^{-2}, the content of carbon and LA133 are 27 wt% and 10 wt% respectively in the cathode.

The density of the materials can be found in Table S1.
